# Viral suppression in adults on efavirenz- or dolutegravir-based antiretroviral therapy in Mopani District, South Africa

**DOI:** 10.4102/sajhivmed.v26i1.1718

**Published:** 2025-08-29

**Authors:** Christine Njuguna, Christina Maluleke, Natasha Davies, Lucia Hans, Barry Mutasa, Kate Rees

**Affiliations:** 1 Anova Health Institute, Johannesburg, South Africa; 2 National Priority Programme, National Health Laboratory Service, Johannesburg, South Africa; 3 Department of Molecular Medicine and Haemotology, Faculty of Health Sciences, University of the Witwatersrand, Johannesburg, South Africa; 4 Department of Community Health, Faculty of Health Sciences, University of the Witwatersrand, Johannesburg, South Africa

**Keywords:** dolutegravir, viral suppression, low-level viremia, TLD, rural health

## Abstract

**Background:**

Dolutegravir- has superior viral suppression compared to efavirenz-based antiretroviral therapy (ART). However, there are limited programmatic data on suppression in rural areas of South Africa.

**Objectives:**

We aimed to compare 6- and 12-month viral suppression of dolutegravir and efavirenz regimens and determine factors available in TIER.Net (the national electronic database for HIV and tuberculosis care) associated with suppression.

**Method:**

We conducted a retrospective cohort study using Mopani District programme data from TIER.Net. Clients aged ≥ 15 years initiated on tenofovir-lamivudine-dolutegravir (TLD) or tenofovir-emtricitabine-efavirenz (TEE) between 01 October 2021 and 31 March 2023, with ≥ 150 days in care, were included. We analysed 6- and 12-month suppression proportions and factors associated with suppression using logistic regression.

**Results:**

A total of 472 clients on TEE and 944 on TLD were included. Six-month viral loads were available for 47.7% (225/472) of TEE and 57.4% (542/944) of TLD clients. Six-month suppression (< 50 copies/mL) was 65.5% (355/542) for TLD and 53.8% (121/225) for TEE (*P* = 0.002). TLD was associated with increased odds of suppression at 6 months (adjusted odds ratio [aOR] 1.6; 95% CI: 1.1–2.2). At 12 months, viral loads were available for 60.7% (573/944) of TLD and 56.1% (265/472) of TEE clients. Twelve-month suppression (< 50 copies/mL) was 70.0% (401/573) for TLD and 68.3% (181/265) for TEE with no statistically significant differences between TEE and TLD clients. Low-level viraemia (50 copies/mL – 999 copies/mL) at 12 months was 25.0% for TLD and 20.8% for TEE.

**Conclusion:**

TLD showed improved suppression compared to TEE at 6 but not 12 months. The high proportion of clients with low-level viraemia is concerning. All clients, regardless of regimen, need evaluation for adherence support.

**What this study adds:** Tenofovir-lamivudine-dolutegravir was associated with higher rates of early viral suppression versus TEE but low-level viraemia was common with both regimens. Enhanced adherence support to optimise viral suppression is needed.

## Introduction

South Africa, with an estimated 7.8 million people living with HIV (PLHIV) in 2022, has the largest global population of PLHIV.^1^ The country aims to reach the 95-95-95 HIV targets by 2030.^2^ However, South Africa is lagging on the second and third 95 with 75% (5.8m) of those who know their HIV status accessing antiretroviral therapy (ART), and 91% (5.3m) of those accessing ART having viral suppression < 1000 copies/mL.^3^ Viral suppression is crucial because it reduces the risk of onward transmission^4^ and morbidity and mortality.^5^

The recommended first-line ART regimen in PLHIV in South Africa from 2014 to 2019 was the fixed-dose combination of tenofovir-emtricitabine-efavirenz (TEE).^6^ However, due to concerns about efavirenz’s side effects and low genetic barrier to resistance, the WHO in 2018^7^ recommended tenofovir and lamivudine or emtricitabine, and dolutegravir (TLD) as the preferred first-line regimen for adolescents and adults, and later for all women regardless of childbearing status.^8,9^ South Africa’s current ART guidelines align with these recommendations, recommending TLD as the preferred first-line regimen since 2019.^10^ However, the transition to TLD was slow in some areas, with delayed rollout of training and early reports of teratogenicity^11^ leading to provider reluctance to initiate women of childbearing potential on dolutegravir. In June 2021, a national Department of Health (DoH) circular stated that women of childbearing potential were now eligible for TLD,^12^ but it is likely that dissemination and implementation of this circular took some time.

Studies have shown that dolutegravir-based regimens have superior viral suppression rates compared to efavirenz-based regimens.^13,14^ Dolutegravir has additional benefits of lower cost, a high genetic barrier to resistance, and better tolerability.^14,15^

Programmatic data from South Africa on viral suppression among clients on dolutegravir versus efavirenz-based regimens are limited. However, studies from sub-Saharan Africa have consistently shown superior 12-month viral suppression (< 50 copies/mL) with dolutegravir regimens. For instance, a Tanzanian study reported viral suppression of 85% with dolutegravir compared to 73% with efavirenz.^16^ Other studies from sub-Saharan African countries reported high suppression rates (84% – 98%) among treatment-experienced individuals transitioning from a non-nucleoside reverse transcriptase inhibitor (NNRTI) regimen to dolutegravir.^17,18,19,20^ A South African study reported that dolutegravir was associated with a higher relative risk of 12-month suppression.^21^

Anova Health Institute, a supporting partner for DoH, funded by the United States (US) President’s Emergency Plan for AIDS Relief (PEPFAR) and the US Agency for International Development (USAID), supported healthcare workers with HIV and tuberculosis training and mentoring and provided ART programmatic support in rural Mopani District, Limpopo province. Programmatic data from South Africa comparing viral suppression in adult clients on TLD and TEE is primarily from urban settings. Urban and rural settings differ in ways that contribute to viral suppression rates, for example education and income levels, distance to clinics, and access to higher levels of care. Little is known about viral suppression with TLD in rural South Africa. The findings of this study could help inform policymakers and clinicians about the management of PLHIV on ART.

The aims of this study were: (1) To determine the 6- and 12-month viral load suppression proportions of clients initiated on TLD compared to TEE, and (2) to measure the association between viral load suppression and available demographic and clinical variables.

## Research methods and design

### Study design

This was a retrospective cohort study.

### Setting

The study was conducted in Mopani District in Limpopo province, South Africa. Mopani District has a predominantly rural (81%) population of approximately 1 372 000. There are an estimated 570 000 PLHIV in Limpopo province, with a prevalence of 8.9%, one of the lowest in South Africa.^22^ In Mopani, achievement of 95-95-95 targets was 93-70-93 in July 2024.^23^

### Study population

#### Inclusion criteria

We included all clients aged 15 years and above initiated on TLD or TEE as a first-line regimen between 01 October 2021 and 31 March 2023, and who had been in care for at least 150 days since ART initiation at the time of data extraction. When TLD was introduced, following the 2019 guidelines update, as the preferred 1st line regimen, clients were eligible to initiate TLD if they were 10 years or older and weighed 35 kg or more. We restricted our analysis to adults above 15 years, who would have all been eligible to initiate TLD, based on these criteria (except in the rare case of severe underweight or stunting).^24^

### Sampling strategy

We extracted data from TIER.Net, a national electronic database containing information on demographic and clinical variables of PLHIV.^25^ A total of 8955 clients met the inclusion criteria, 472 on a TEE regimen and 8483 on a TLD regimen. We used exact matching in order to increase the efficiency of the analysis and partially remove the confounding effect of the matching variables, without introducing additional selection bias.^26^ We aimed to sample at least 944 clients on TLD to have an approximate ratio of 1:2.

We used matching to create groups with similar sex, age (categorised as 15–24 years, 25–49 years, ≥ 50 years) and year of ART start (2021, 2022, 2023) (18 groups created in total). We then randomly sampled 944 clients on TLD using STATA version 18 (College Station, Texas, US). The final sample used in this analysis was 1416 records.

### Study variables

#### Outcome variables

The outcome variables were viral suppression at 6 and 12 months. A viral load cut-off < 50 copies/mL was used to indicate viral suppression. An additional viral load cut-off < 1000 copies/mL was used to measure low-level viraemia (defined as 50 copies/mL – 999 copies/mL) at the 6- and 12-month time points. According to the DoH guidelines, viral load monitoring should have been conducted twice, at 6 and at 12 months, if the client was suppressed.^27^ Additional viral loads were required for raised viral load results. A 6-month viral load was defined as a viral load taken between 5 and 7 months from ART initiation date. A 12-month viral load was defined as a viral load taken between 11 and 13 months from ART initiation date.

#### Exposure variables

We included demographic, clinical and treatment variables. The demographic variables were age at last visit, sex, and sub-district. The clinical variables were baseline CD4 count, tuberculosis treatment at ART initiation, viral load values, and viral load dates done during the study period. Treatment variables were ART regimen, year of ART initiation, and enrolment into differentiated models of care (DMOC).

### Statistical analysis

Simple proportions were computed to describe viral suppression at 6 and 12 months in clients on TEE and TLD regimens. The differences in proportions were measured using Chi-squared statistic or Fischer’s exact test.

Bivariable and multivariable logistic analysis was conducted. Exposure variables were selected a priori based on their association with the outcome variable as well as availability on TIER.Net. We reported adjusted odds ratios, their corresponding 95% confidence intervals and *P*-values. A *P* < 0.05 was considered statistically significant. All data were analysed using STATA software version 18 (College Station, Texas, US).

### Ethical considerations

Ethical approval was obtained from the Human Sciences Research Council Research Ethics Committee (reference number: REC 3/22/08/18). This study used anonymised TIER.Net data which are routinely collected at healthcare facilities for monitoring purposes; therefore, individual consent was not required.

## Results

A total of 8955 clients started ART between 01 October 2021 and 31 March 2023 and met all inclusion criteria. After sampling using a 1:2 ratio, a total sample size of 1416 eligible clients was included in the analysis.

### Baseline characteristics

There were 944 and 472 clients initiated on dolutegravir and efavirenz first-line ART regimens. Most clients on TLD and TEE (*P* = 1.000) were aged between 25 and 49 years and were female (Table 1). Most clients were enrolled from clinics in the Greater Tzaneen sub-district. The proportion of clients with advanced HIV disease (CD4 < 200) was 20.0% on TLD and 15.5% on TEE (*P* < 0.001). The proportion of clients enrolled in DMOC at the time of data extraction was 38.5% for TLD and 33.1% for TEE (*P* = 0.047) (Table 1).

**TABLE 1:** Baseline characteristics of clients on tenofovir-lamivudine-dolutegravir and tenofovir-emtricitabine-efavirenz regimens.

Variable	TLD regimen (*n* = 944)		TEE regimen (*n* = 472)		*P*
	*n*	%	*n*	%	
**Age category (years)**	-	-	-	-	1.000
15–24	130	13.77	65	13.77	
25–49	720	76.27	360	76.27	
≥ 50	94	9.96	47	9.96	
**Sex**	-	-	-	-	1.000
Female	766	81.14	383	81.14	
Male	178	18.86	89	18.86	
**Baseline CD4 category (mm^3^)**	-	-	-	-	0.000
< 200	189	20.02	73	15.47	
≥ 200	365	38.67	152	32.20	
Missing	390	41.31	247	52.33	
**Anti-tuberculosis treatment at ART initiation**	-	-	-	-	0.000
No	414	43.86	225	47.67	
Unknown	484	51.27	173	36.65	
Yes	46	4.87	74	15.68	
**Enrolled in DMOC at time of data extraction**	-	-	-	-	0.047
No	581	61.55	316	66.95	
Yes	363	38.45	156	33.05	
**Sub-district**	-	-	-	-	0.000
Ba-Phalaborwa	138	14.62	47	9.96	
Greater Giyani	203	21.50	135	28.60	
Greater Letaba	188	19.92	107	22.67	
Greater Tzaneen	355	37.61	119	25.21	
Maruleng	60	6.36	64	13.56	
**ART start date (years)**	-	-	-	-	1.000
2021	360	38.14	180	38.14	
2022	540	57.20	270	57.20	
2023	44	4.66	22	4.66	

TLD, tenofovir-lamivudine-dolutegravir; TEE, tenofovir-emtricitabine-efavirenz; ART, antiretroviral therapy; DMOC, differentiated models of care.

### Programme losses

At the end of the follow-up period, 85.3% (805/944) and 80.5% (380/472) of clients on TLD and TEE remained in care: 0.2% (2/944) and 1.1% (5/472) had died in the TLD and TEE group, 4.7% (44/944) and 5.3% (25/472) on TLD and TEE were lost to follow-up, and 9.9% (93/944) and 13.1% (62/472) on TLD and TEE were transferred out.

### Six-month and twelve-month viral suppression

At 6 months, 57.4% (542/944) of clients on TLD and 47.7% (225/472) of clients on TEE had a viral load done. At 12 months, 60.7% (573/944) and 56.1% (265/472) of those on TLD and TEE regimens had viral loads done (Table 2).

**TABLE 2:** 6- and 12-month viral suppression proportions for clients on dolutegravir and efavirenz-based regimens.

ART regimen	*n*	Viral suppression number	Viral suppression proportion		Viral suppression percentage difference	*P*
			%	95% CI		
**6-month viral load suppression (< 50 copies/mL)**	-	-	-	-	11.72	0.002**
TLD regimen	542	355	65.50	61.33–69.50	-	-
TEE regimen	225	121	53.78	47.03–60.43	-	-
**12-month viral load suppression (< 50 copies/mL)**	-	-	-	-	1.68	0.623
TLD regimen	573	401	69.98	66.05–73.71	-	-
TEE regimen	265	181	68.30	62.33–73.86	-	-
**6-month viral load suppression (< 1000 copies/mL)**	-	-	-	-	4.87	0.015*
TLD regimen	542	513	94.65	92.41–96.39	-	-
TEE regimen	225	202	89.78	85.06–93.41	-	-
**12-month viral load suppression (< 1000 copies/mL)**	-	-	-	-	5.88	0.002**
TLD regimen	573	544	94.94	92.81–96.58	-	-
TEE regimen	265	236	89.06	84.66–92.55	-	-

TLD, tenofovir-lamivudine-dolutegravir; TEE, tenofovir-emtricitabine-efavirenz; ART, antiretroviral therapy.

* , *P* < 0.05;

** , *P* ≤ 0.01.

At 6 months 65.5% (355/542; 95%CI:61.3–69.5) of those on TLD compared to 53.8% (121/225; 95%CI: 47.0–60.4) of those on TEE were suppressed at < 50 copies/mL (proportion difference 11.7%; *P* = 0.002) (Table 2). For those on TLD, 94.7% (513/542; 95%CI:92.4–96.4) attained a viral load < 1000 copies/mL compared to 89.8% (202/225; 95%CI: 85.1–93.4) on TEE (proportion difference 4.9%; *P* = 0.015) (Table 2). Overall, at 6 months, 31.2% (239/767) of clients with a viral load done had low-level viraemia (50–999 copies/mL). Low-level viraemia was 29.2% (158/542) and 36.0% (81/225) for TLD and TEE. At 12 months, 60.7% (145/239) of those with low-level viraemia at 6 months had a further viral load done. Of these, 54.5% (79/145) suppressed to below 50 copies/mL, 36.6% on TLD and 17.9% on TEE (data not shown).

At 12 months, 70.0% (401/573; 95%CI: 66.1–73.7) of those on TLD compared to 68.3% (181/265; 95%CI: 62.3–73.9) on TEE (proportion difference 1.7%; *P* = 0.623) were suppressed at < 50 copies/mL (Table 2). For those on TLD, 94.9% (544/573; 95%CI: 92.8–96.6) attained a viral load < 1000 copies/mL compared to 89.1% (236/265; 95%CI: 84.7–92.6) on TEE (proportion difference 5.9%; *P* = 0.002) (Table 2). At 12 months, low-level viraemia (50–999 copies/mL) was 25.0% and 20.8% for TLD and TEE (data not shown).

### Factors associated with 6- and 12-month viral suppression

At 6 months, the following factors were associated with increased odds of viral suppression < 50 copies/mL: being on TLD and being enrolled in DMOC (Table 3).

**TABLE 3:** Multivariable logistic regression of the predictors of viral load suppression (< 50 copies/mL) at 6 and 12 months for dolutegravir-based regimens compared to efavirenz-based antiretroviral therapy regimens.

Variable	6-month viral suppression (< 50 copies/mL) *n* = 767						12-month viral suppression (< 50 copies cut-off) *n* = 838					
	Unadjusted OR	95% CI	*P*	Adjusted OR	95% CI	*P*	Unadjusted OR	95%CI	*P*	Adjusted OR	95%CI	*P*
**ART regimen**												
TEE regimen	1	-	-	1	-	-	1	-	-	1	-	-
TLD regimen	1.63	1.19–2.24	0.002	1.56	1.11–2.21	0.011*	1.08	0.79–1.48	0.623	0.92	0.65–1.30	0.642
**Age category (years)**												
15–24	1	-	-	1	-	-	1	-	-	1	-	-
25–49	1.14	0.74–1.74	0.557	1.10	0.70–1.73	0.684	1.65	1.08–2.52	0.022	1.71	1.09–2.70	0.020*
≥ 50	0.72	0.40–1.31	0.280	0.70	0.36–1.37	0.299	1.41	0.77–2.57	0.266	1.56	0.80–3.05	0.193
**Sex**												
Female	1	-	-	1	-	-	1	-	-	1	-	-
Male	0.67	0.47–0.97	0.032	0.74	0.49–1.12	0.157	0.63	0.44–0.91	0.015	0.61	0.40–0.92	0.020*
**Tuberculosis treatment at ART initiation**												
No	1	-	-	1	-	-	1	-	-	1	-	-
Unknown	1.13	0.83–1.54	0.424	1.08	0.78–1.49	0.661	1.15	0.84–1.56	0.386	1.08	0.78–1.51	0.638
Yes	0.65	0.37–1.12	0.121	0.94	0.51–1.71	0.829	0.86	0.51–1.44	0.560	0.87	0.49–1.55	0.639
**DMOC enrolment**												
No	1	-	-	1	-	-	1	-	-	1	-	-
Yes	2.61	1.91–3.58	0.000	2.56	1.85–3.53	0.000***	3.10	2.24–4.30	0.000	3.27	2.34–4.58	0.000***
**Sub-district**												
Ba-Phalaborwa	1	-	-	1	-	-	1	-	-	1	-	-
Greater Giyani	1.03	0.60–1.75	0.924	0.96	0.55–1.68	0.898	0.94	0.56–1.57	0.799	0.81	0.47–1.39	0.438
Greater Letaba	1.18	0.68–2.05	0.548	1.11	0.62–1.99	0.727	0.83	0.49–1.39	0.473	0.68	0.39–1.18	0.171
Greater Tzaneen	1.07	0.64–1.80	0.783	1.00	0.58–1.70	0.992	0.92	0.56–1.50	0.737	0.84	0.50–1.40	0.494
Maruleng	1.23	0.65–2.36	0.524	1.09	0.55–2.18	0.797	0.66	0.36–1.21	0.182	0.53	0.28–1.01	0.053
**ART start year**												
2021	1	-	-	1	-	-	1	-	-	1	-	-
2022	0.78	0.57–1.07	0.126	0.90	0.64–1.25	0.515	1.11	0.82–1.51	0.504	1.35	0.97–1.86	0.075
2023	0.85	0.43–1.66	0.634	1.18	0.58–2.40	0.639	1.41	0.28–7.13	0.676	2.11	0.40–11.19	0.379

ART, antiretroviral therapy; TEE, tenofovir-emtricitabine-efavirenz; TLD, tenofovir-lamivudine-dolutegravir; DMOC, differentiated models of care.

* , for adjusted odds ratios: *P* ≤ 0.05;

** , *P* < 0.01;

*** , *P* < 0.001.

However, at 12 months, viral suppression < 50 copies/mL was not associated with being on a TLD regimen after adjusting for age, sex, tuberculosis treatment at ART initiation, being enrolled in DMOC, sub-district, and year of ART initiation (Table 3).

Factors associated with increased odds of viral suppression < 50 copies/mL at 12 months were: clients aged 25–49 years versus 15–24 years and being enrolled in DMOC. Male sex was associated with decreased odds of viral suppression < 50 copies/mL (Table 3).

A 6-month viral load < 1000 copies/mL was associated with being on TLD, enrolled in DMOC, and aged 25–49 years compared to 15–24 years (Table 4).

**TABLE 4:** Multivariable logistic regression model of association between viral load < 1000 copies/mL at 6 and 12 months for dolutegravir-based regimens compared to efavirenz-based antiretroviral therapy regimens.

Variable	6-month viral suppression (< 1000 copies/mL) *n* = 767						12-month viral suppression (< 1000 copies cut-off) *n* = 838					
	Unadjusted OR	95% CI	*P*	Adjusted OR	95% CI	*P*	Unadjusted OR	95% CI	*P*	Adjusted OR	95% CI	*P*
**ART regimen**												
TEE regimen	1	-	-	1	-	-	1	-	-	1	-	-
TLD regimen	2.01	1.14–3.56	0.016	1.86	0.99–3.51	0.055	2.31	1.35–3.94	0.002	2.24	1.25–4.02	0.007**
**Age category (years)**												
15–24	1	-	-	1	-	-	1	-	-	1	-	-
25–49	3.25	1.71–6.19	0.000	3.21	1.56–6.62	0.002**	3.02	1.64–5.57	0.000	2.97	1.51–5.87	0.002**
≥ 50	2.65	0.93–7.59	0.069	2.95	0.87–10.04	0.084	7.92	1.77–35.35	0.007	6.89	1.42–33.48	0.017*
**Sex**												
Female	1	-	-	1	-	-	1	-	-	1	-	-
Male	0.70	0.37–1.36	0.295	0.59	0.27–1.28	0.179	1.16	0.56– 2.42	0.692	0.93	0.41–2.15	0.874
**Tuberculosis treatment at ART initiation**												
No	1	-	-	1	-	-	1	-	-	1	-	-
Unknown	1.29	0.71–2.34	0.411	1.09	0.57–2.08	0.800	1.26	0.72–2.19	0.416	1.10	0.60–2.01	0.758
Yes	0.73	0.28–1.86	0.504	0.98	0.35–2.78	0.970	1.57	0.53–4.61	0.414	1.49	0.46–4.87	0.505
**DMOC enrolment**												
No	1	-	-	1	-	-	1	-	-	1	-	-
Yes	9.50	3.39–26.64	0.000	9.03	3.19–25.57	0.000***	9.04	3.57–22.86	0.000	9.50	3.69–24.49	0.000***
**Sub-district**												
Ba-Phalaborwa	1	-	-	1	-	-	1	-	-	1	-	-
Greater Giyani	1.36	0.44–4.20	0.590	1.27	0.40–4.06	0.690	1.60	0.56–4.53	0.378	1.72	0.58–5.08	0.324
Greater Letaba	0.76	0.26–2.21	0.613	0.76	0.25–2.36	0.638	0.76	0.30–1.93	0.568	0.73	0.27–1.97	0.539
Greater Tzaneen	0.79	0.29–2.17	0.643	0.78	0.27–2.25	0.648	0.97	0.39–2.42	0.955	0.90	0.35–2.30	0.819
Maruleng	0.96	0.27–3.46	0.950	0.95	0.24–3.72	0.940	0.43	0.16–1.16	0.095	0.39	0.13–1.14	0.086
**ART start year**												
2021	1	-	-	1	-	-	1	-	-	1	-	-
2022	0.63	0.33–1.18	0.147	0.83	0.42–1.65	0.597	1.15	0.67–1.99	0.606	1.35	0.75–2.43	0.313
2023†	2.26	0.29–17.65	0.437	3.26	0.40–26.50	0.268	-	-	-	-	-	-

ART, antiretroviral therapy; TEE, tenofovir-emtricitabine-efavirenz; TLD, tenofovir-lamivudine-dolutegravir; DMOC, differentiated models of care.

† , Odds ratio omitted from model as all observations for ART start year (2023) predicted success for the binary outcome variable, viral suppression < 1000 copies/mL.

* , for adjusted odds ratios: *P* ≤ 0.05;

** , *P* < 0.01;

*** , *P* < 0.001.

Factors associated with 12-month viral suppression < 1000 copies/mL were being on TLD, aged 25–49 years or ≥ 50 years compared to those aged 15–24 years, and being enrolled in DMOC (Table 4).

## Discussion

We found increased odds of viral suppression < 50 and < 1000 copies/mL in people on TLD compared to those on TEE at 6 months. At 12 months, however, TLD was not associated with suppression < 50 copies/mL, although it was associated with viral load < 1000 copies/mL. Globally, research clearly demonstrates that dolutegravir-based regimens result in superior viral suppression compared to EFV-based regimens. However, this study, which focuses on outcomes in a rural district in South Africa, found that even though the majority of clients are accessing dolutegravir-based first line treatment, virological non-suppression remains common. Considering dolutegravir’s superior efficacy when taken optimally, this finding highlights the ongoing challenges around supporting sustained adherence and retention in care within resource-constrained contexts. Further research is needed to identify evidence-based support strategies that effectively address widespread adherence issues to improve viral suppression rates. Additionally, we would advocate for wider access to affordable drug exposure testing to support clinicians to differentiate between adherence-driven non-suppression versus the possible emergence of dolutegravir resistance within the context of frequent, and often prolonged, periods of suboptimal adherence and resultant ongoing viral replication.

Our findings of improved viral suppression with TLD are similar to published programmatic data.^16,17,18,21^ However, our suppression rates of 66% and 70% at 6 and 12 months on TLD were lower than those reported previously. For example, a study from rural Tanzania among treatment-naïve adolescent and adult clients reported 12-month viral suppression (< 50 copies/mL) of 85% with dolutegravir-based regimens and 73% with an NNRTI-based regimen.^16^ A study from a South African urban district (eThekwini) reported 12-month viral suppression of 83% in an adult cohort of 12 000 clients initiated on dolutegravir compared to 81% in those on NNRTI regimens.^21^ It is important to note that our study was conducted using programmatic data, in a rural setting having scaled the use of TLD. Clients in rural areas may have limited access to health facilities due to long distances to clinics, or the programme may be too large and lack the capacity for adequate adherence support, both of which can negatively impact client outcomes.

In multivariable logistic regression, being on TLD was associated with increased odds of viral suppression < 50 copies/mL and viral load < 1000 copies/mL. Another rural cohort from Tanzania reported similar results.^16^ Older age (25–49 years compared to clients aged 15–24 years) and being enrolled in DMOC increased the odds of viral suppression. Clients in DMOC are typically stable, with suppressed viral loads when enrolled. Hence, the association that is observed may be due to selection bias. Interestingly, there was no association between sex and viral suppression < 50 copies/mL at 6 months; however, at 12 months being male was significantly associated with decreased odds of viral suppression. This finding suggests that male clients may require additional adherence support with longer ART duration.

We found high proportions of low-level viraemia (50–999 copies/mL), with approximately 21% and 25% of clients on efavirenz and dolutegravir-based regimens experiencing this at 12 months. Interestingly, although TLD was not associated with suppression < 50 copies/mL at 12 months, it was associated with viral load < 1000 copies/mL. This may suggest that TLD in this setting protects against progression to virological failure, despite not achieving complete suppression to below 50 copies. Poor adherence is a common cause of low-level viraemia in PLHIV.^28^ Our data also showed that just over half (54.5%) of clients re-suppressed at 12 months after initial low-level viraemia at 6 months. This underscores the importance of strengthening adherence measures even in people who are still retained in care and on TLD, particularly in men, over time. In this rural setting, another explanation might be the concurrent use of traditional or herbal supplements that may have unknown interactions with dolutegravir, potentially impacting on absorption or increasing metabolism, thereby decreasing its bioavailability and efficacy. One South African study showed that PLHIV on a dolutegravir-based ART regimen taking concomitant herbal or traditional medicines had decreased odds of viral suppression (< 50 copies/mL).^29^

A number of studies have consistently shown an association between low-level viraemia and an increased risk of viral non-suppression^30,31^ progressing to virologic failure.^31^ Nonetheless, our rates of virological failure, based on viral load ≥ 1000 copies/mL, were low: 5% for TLD and 10% for TEE regimens at both 6 and 12 months. This cut-off is considered clinically important for identifying those where suboptimal adherence requires additional support to avoid confirmed treatment failure and emergence of HIV drug resistance.^32^ It is important to note that we only looked at viral suppression to 12 months and the risk of confirmed treatment failure with emergence of drug resistance in this unsuppressed group may increase over time if suppression cannot be achieved through provision of intensified support.

Viral load completion at set time points was low, ranging from 47.7% to 57.4% at 6 months and 56.1% to 60.7% at 12 months. This is concerning as it may suggest that clients without viral load monitoring had missed visits or interrupted treatment, so there is a possibility that suppression rates in those not monitored may be lower, resulting in overall poorer individual and programme outcomes. Our definitions were stricter than those used in programme monitoring. The literature on viral load completion rates is mixed and varies based on the geographic setting. Our findings were similar to data from a rural cohort in KwaZulu-Natal, South Africa,^33^ as well as data from another rural HIV cohort from Tanzania.^16^ In contrast, two other studies from urban settings in Lesotho and South Africa reported higher viral load completion of 85.8% and 88.9% at 12 months.^20,21^ Distance to the clinic is one factor that is likely to affect viral load completion and viral suppression, but there are several others that are likely to be different in rural areas, where resources are usually more limited. These may include training of health care workers (HCWs) on viral load monitoring, organisational and management factors, incorrect recording and long waiting times.^34^ Due to the limited scope of this analysis, the authors are unable to comment on the important issues pertaining to programmatic performance emerging from the data set. In particular, slow transition to TLD as first line ART, and low viral load coverage at 6 and 12 months on treatment were noted across the district. Future research is needed to explore the underlying drivers of suboptimal ART guidelines implementation regarding use of optimized ART regimens and comprehensive monitoring to confirm treatment adherence and viral suppression. Should such studies be undertaken, it would be valuable to conduct an analysis across urban, periurban and rural districts to understand any differences in implementation challenges. Where implementation gaps are identified, implementation science studies are needed to develop interventions that successfully address context specific barriers to improve programmatic performance and clinical outcomes. Our findings also suggest that research exploring the impact of community-based point-of-care viral load monitoring may be of value.

Strengths of this study include our use of routinely collected data from an electronic database, TIER.Net, which enhanced study efficiency while still providing a robust data set that enabled us to measure client outcomes. The study had some limitations. First, the sample size was small, due to the smaller number of clients being initiated on efavirenz-based regimens during the study period. Second, the viral load completion rates at set time points in this study were lower than expected. This may have led to under- or over-estimation of the viral load suppression proportions in both groups. Third, the baseline CD4 count and pregnancy variables had a lot of missing data; thus, we could not include them in our logistic regression analysis. Furthermore, laboratory-related factors such as inappropriate sample collection, inappropriate sample transportation, or test kit sensitivity could not be measured. Fourth, data on clinic visits or pharmacy refill data were not collected; therefore, we were unable to measure retention or adherence. Fifth, the two groups were different, with the TLD group having more advanced HIV disease (CD4 < 200 cells/μL), so the effects of TLD on viral suppression may be underestimated.

## Conclusion

Tenofovir-lamivudine-dolutegravir showed improved viral suppression (< 50 copies/mL) compared to TEE at 6 months and < 1000 copies/mL at 12 months. This study adds to the evidence base that supports the use of TLD as first-line ART in large-scale programmatic settings. However, the low viral load monitoring and the proportion of clients with low-level viraemia even on TLD is concerning. All clients, regardless of ART regimen, need evaluation for intensified adherence support, and interventions to enhance viral load monitoring should be considered. Programmes may need adaptations to address the complexities of managing clients in a rural setting where outcomes may not be as good.
